# Early application of topical antibiotic powder in open-fracture wounds: A strategy to prevent biofilm formation and infections

**DOI:** 10.1097/OI9.0000000000000091

**Published:** 2020-10-12

**Authors:** Kimberly M. Burbank, Steven G. Schauer, Robert A. De Lorenzo, Joseph C. Wenke

**Affiliations:** a US Army Institute of Surgical Research; b Brooke Army Medical Center, JBSA Fort Sam Houston; c 59th Medical Wing, JBSA Lackland, TX; d Uniformed Services University of the Health Sciences, Bethesda, MD; e Department of Emergency Medicine, University of Texas Health Sciences Center at San Antonio, San Antonio, TX.

**Keywords:** antibiotic, fracture, infection, open, prophylaxis

## Abstract

Despite meticulous surgical care and systemic antibiotics, open fracture wounds have high rates of infection leading to increased morbidity. To reduce infection rates, orthopaedic surgeons may administer local antibiotics using various carriers that may be ineffective due to poor antibiotic release from carriers, subsequent surgery to remove nondegradable carriers, and mismatch between release kinetics and material degradation. Biofilms form rapidly as bacteria that are within the wound multiply quickly and transform from the antibiotic-susceptible planktonic phenotype to the antibiotic-tolerant biofilm phenotype. This tolerance to antibiotics can occur within hours. Currently, local antibiotics are placed in the wounds using a carrier such as polymethylmethacrylate beads; however, this occurs after surgical debridement that can be hours to even a day after initial injury allowing bacteria enough time to form a biofilm that makes the antibiotic containing polymethylmethacrylate beads less effective. In contrast, emerging practices in elective surgical procedures, such as spine fusion, place antibiotic powder (e.g. vancomycin) in the wound at the time of closure. This has been shown to be extremely effective, presumably because of the very small-time period between potential contamination and local antibiotic application. There is evidence that suggests that the ineffectiveness of local antibiotic use in open fractures is primarily due to the delay in application of local antibiotics from the time of injury and propose a concept of topical antibiotic powder application in the prehospital or emergency department setting.

## 1 Introduction

Open fracture wounds are exposed to contaminated environments which makes them at high risk of infection^[1]^ Major complications such as infections are the primary predictors of poor outcome in patients who suffered traumatic injury^[2]^ and are known to complicate recovery, increase morbidity, and in rare cases even cause mortality.^[3]^ The current mainstays for preventing infection in open fractures are thorough debridement of the wound and use of systemic antibiotics. Despite this, many wounds still become infected. To decrease infection rates in open fracture wounds, local antibiotics have been used in surgery as an adjunct to systemic antibiotics.^[4,5]^ Unlike systemic antibiotics, high concentrations of antibiotic are capable with use of local depots, which are believed to be more effective. Despite the theoretical advantages and studies that demonstrated effectiveness in preclinical models, there is little clinical evidence that local antibiotics improve outcomes of open fractures. Most antibiotic carriers used clinically have poor release kinetics of antibiotics and introduce foreign bodies to the wound. These factors may play a role, but they are likely not the primary reason why local antibiotic delivery appears to be relatively ineffective in open fractures.

We propose that the most likely explanation is the timing from injury to application of local antibiotics. Preclinical evidence, elective orthopaedic procedures, and specific clinical studies on open fractures have shown a reduction in infection rates when antibiotics are applied early. This suggests that infection mitigation has more to do with antibiotics reaching the bacteria quickly than the delivery method or type of antibiotic chosen.^[6–9]^ This will be explained with the backdrop of the biofilm theory, which describes how bacteria divide and grow in number until they have reached a certain population density and change from the antibiotic-susceptible planktonic phenotype to the antibiotic-tolerant biofilm phenotype.^[10,11]^ This can occur in as little as a few hours. To effectively take advantage of local antibiotic therapy, the application of local antibiotics must be uncoupled from surgery, which often is not performed until hours or up to a day after injury and contamination of the wound. The use of topical antibiotic powders in the prehospital or emergency department setting will allow high levels of antibiotics at the wound site while not allowing the bacteria to form a biofilm and become recalcitrant to antibiotic therapy.

## 2 Biofilm theory

Specific to open fracture wounds, the biofilm theory explains why the current approach for preventing infection is often unsuccessful. Bacteria enter the wound in a planktonic, antibiotic-susceptible phenotype, attach to the surface of tissue, divide and grow in numbers until they reach a certain population density, and shift into an antibiotic-tolerant biofilm phenotype.^[12]^ These bacteria secrete extracellular polymeric substances that form a matrix of protein, polysaccharide, and extracellular DNA, which provides a barrier and protects them from being phagocytosed by host immune cells. Some of the cells, particularly the ones deep within the sessile community, reduce their metabolism and replication which makes them tolerant to antibiotic therapy. It has been shown that it can take 1000-fold more antibiotic to eradicate persister cells within a biofilm.^[13]^ The exact timing of biofilm formation and maturation depends on many factors such as the amount of initial colonization, environment, and bacteria species and strain. With that said, it has been shown that development of a biofilm occurs as early as 5 hours after inoculation, with maturation of this biofilm by 10 hours.^[14]^ After the systemic or local antibiotic therapy is finished, the persisting bacteria become metabolically active and replicate. This is believed to be one of the major reasons behind the high infection rates in open fracture wounds.^[15]^

## 3 Antibiotic timing

Open fracture wounds are predominantly caused by high-energy mechanisms. The wound is instantly exposed to debris and bacteria from the environment, increasing the probability of infection.^[16]^ The primary methods for infection mitigation and prevention in the treatment of open fracture wounds are antibiotic administration, debridement, irrigation, and wound closure.^[17]^ Intravenous antibiotics is the current practice for open fracture prophylaxis.^[18]^ However blood flow is often compromised in these wounds, which affects the ability for systemic antibiotics to reach the injury site in high enough concentrations.^[17]^ Furthermore, increasing the dose of antibiotics to adequately eradicate bacteria puts the patient at risk for toxicity to nontarget organs (e.g., kidney, liver, etc.).^[19]^ Local antibiotic therapies have provided a way to overcome poor perfusion issues in open fractures, but unfortunately infection still persists.^[20,21]^ We believe this is due to high concentrations of antibiotic not reaching the wound site quickly enough through the currently used local antibiotic delivery methods, which is generally polymethylmethacrylate (PMMA) beads. Overall, clinical evidence for the early administration of antibiotics reducing infections has not been consistent (Table 1).^[1,16,22–25]^ The few clinical studies that have assessed for timing of antibiotic application use an arbitrary 3-hour time point to classify as early or late administration. Lack et al, however, used very early time periods to demonstrate that time from injury to antibiotics is significant. Type III open tibia fractures, which are the most severe grade and likelihood of infection, had an infection rate of 7% with antibiotics administered within an hour and 28% when antibiotics were given beyond 90 minutes.^[24]^ This strongly supports the idea that the earlier the better for antibiotic administration.

**Table 1:**
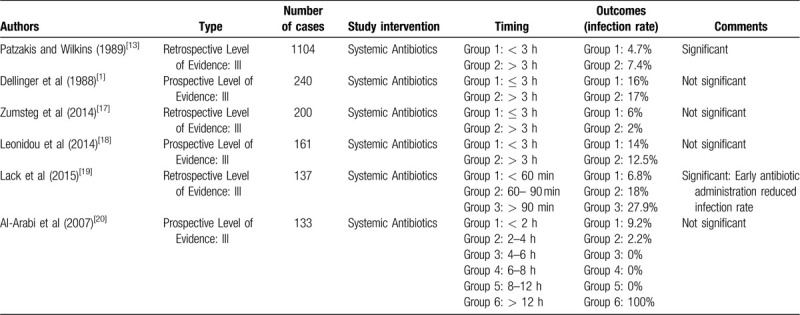
Selected clinical studies reporting the timing of antibiotic delivery in open fractures

Currently, clinical studies are limited, but preclinical studies clearly demonstrate a temporal effect between antibiotic administration and bacterial inoculation. A preclinical study found that antimicrobial timing had a clear effect on the level of bacteria present in a rat femur defect after initial infection. Animals who received antibiotics and surgery 2 hours after injury were found to have no quantifiable levels of bacteria, while the animals who had 24-hour delayed treatment were all infected.^[8]^ These studies support the notion that the closer to injury the antibiotic is administered, the more effective they are at reducing infection.

## 4 Local antibiotic strategies for treating open fractures

Intravenous antibiotics’ potential inability to reach the open-fracture site quickly and in sufficiently high concentrations poses a unique problem. Local antibiotics have been used to address this issue, but infection persists. Currently, the available local antibiotic treatments use antibiotic vehicles such as PMMA cement, chitosan, collagen gauze, and calcium sulfate. With their ability to achieve high concentrations of drug at the wound site, local antibiotics were thought to be a promising adjunct to systemic antibiotics in the treatment of open-fracture wounds, but have demonstrated limited success in clinical studies (Table 2).^[5,20,21,26,27]^ While preclinical data demonstrates the effectiveness of local antibiotics, the clinical evidence supporting this approach is not as robust.^[28]^ The delay of local antibiotic administration is thought to be the contributing factor. PMMA cement is the most frequently used local antibiotic delivery method.^[5,29]^ However, PMMA cement does not biodegrade so a subsequent surgery is needed for removal. Additionally, self-made beads have inconsistent and poor elution characteristics.^[30]^ After the first few weeks, the antibiotics released drop below measurable and therapeutic levels causing the beads to act as foreign bodies on which bacteria colonize.^[31,32]^ This furthers risk of antibiotic resistance and biofilm formation.^[15]^ In light of the biofilm theory, delays in application appear to be the most probable reason for failure.^[9]^ Antibiotic timing is critical to infection mitigation as demonstrated.^[9,16,24]^ PMMA beads are not applied until surgery, which is often hours to a day after the open fracture occurs. This allows the bacteria to develop a biofilm and renders the treatment ineffective against the antibiotic tolerant bacteria. A recent study demonstrating the relationship between the time course of local antibiotic treatment to infection in a preclinical open fracture model supports this idea.^[9]^ In this study, delaying application of antibiotic impregnated PMMA beads from 2 hours after bacteria inoculation to 6 hours resulted in a much higher number of bacteria within the wound. A further delay to 24 hours resulted in even more bacteria within the wounds.

**Table 2:**
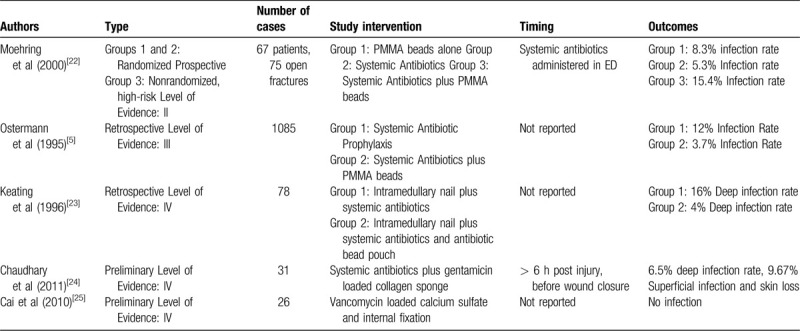
Selected clinical studies of local antibiotic delivery in open fractures

## 5 Direct application local antibiotic powder

**Figure 1:**
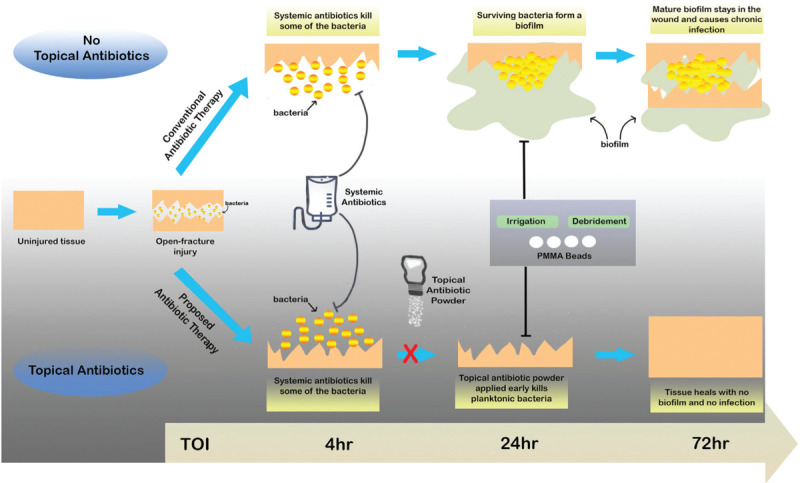
Conventional versus proposed early topical antibiotic administration on development of biofilm infections. Bacterial contamination often occurs at the time of injury (TOI). The conventional antibiotic approach in open fractures uses early systemic antibiotics (typically within 1–3 h) and local antibiotic-impregnated bone cement (typically 12 h to several days). Systemic antibiotics act on planktonic and loosely attached bacteria, but often fail to completely eradicate bacteria. Poor blood flow to the damaged tissue may reduce the concentration of antibiotics that reach the wound site. Surviving bacteria begin developing into the biofilm phenotype, which evade the host immune system, are not completely removed by irrigation and debridement, and become refractory to antibiotics. Local therapy is effective against bacteria because it promotes higher tissue concentrations than IV administration alone. The proposed use of topical antibiotic powder uncouples local therapy from surgery allowing antibiotics to be pushed much earlier. Systemic antibiotics will still be used and antibiotic impregnated PMMA beads may be needed for space maintenance in defects until bone grafting occurs.

The application of antibiotics directly into the wound site circumvents many of the delayed application issues posed by PMMA beads and other local antibiotic delivery methods, while retaining benefit of high local drug concentrations. The direct application of vancomycin powder has been primarily studied and supported in the elective spinal surgery setting.^[33–36]^ Other preclinical and clinical studies have shown a benefit to the application of vancomycin powder in reducing infection rates.^[7,37]^ Vancomycin powder can be applied early and directly into the wound, which eliminates previously noted delays until surgery. Additionally, the direct application into the wound circumvents the lack of blood flow problem posed by many open fractures, which reduces the effectiveness of systemic antibiotics. The early application of antibiotic powder is particularly important in order to reach the wound site in high concentrations before biofilm formation (Fig. 1).^[38]^ This approach may overcome some of the limitations of systemic administration of vancomycin: renal injury, hypersensitivity, anaphylaxis, and possible selection of multidrug-resistant gram-negative infections. These concerns have remained unsubstantiated.^[39]^ A retrospective cohort study showed no adverse clinical outcomes or wound complications attributed to the local application of vancomycin powder in 911 thoracic and lumbar spinal fusions.^[40]^ Further studies also did not see side effects or systemic issues from the topical application of vancomycin powder, adding to its appeal as a safe treatment option for open-fracture wounds.^[41,42]^ A recent preclinical study in a rat model corroborates the clinical findings. When applied early (6 hours after bacterial contamination), the vancomycin powder eradicated the infection within the wound and the levels of antibiotic within the blood were extremely low. However, if the local therapy was delayed until 24 hours, the vancomycin powder did not reduce the amount of bacteria within the wound.^[7]^ This is likely due to the increased concentration of bacteria in the wound and biofilm formation. Although the concept of applying an antibiotic powder directly into the wound appears foreign or unconventional, it has been done previously. In World War II, American soldiers were given first-aid kits containing sulfa powder and were instructed to sprinkle the powder on open wounds.^[43]^ However, these sulfonamide drugs were replaced when the introduction of penicillin proved to be a more potent antibiotic therapy, and the method of applying antibiotic powder topically was left behind.^[44]^ More than 75 years later, locally applied antibiotic powder has potential to reemerge as an adjunct treatment for open-fracture wounds. Additionally, vancomycin appears to be one of the safest drugs when it comes to host cell toxicity^[45]^ and does not impair bone healing in a gap defect rodent model.^[46]^

## 6 Ongoing and emerging clinical trials

Clinical trials using the vancomycin powder are underway that may provide higher levels of evidence related to our proposed use. The Major Extremity Trauma Research Consortium's Vancomycin Study (NCT# 02227446) and the University of Tennessee's Local Application of Vancomycin Powder in Grade I-IIIA Open Fractures (NCT# 02400112) are analyzing the postoperative rates of infection after the vancomycin powder is placed at the time of surgical closure. We will study this concept in a multicenter clinical trial,^[47]^ “Placement Of antibiotic powder in Wounds During the Emergency Room” (POWDER) (NCT# 03765567), will be assessing patient-centered outcomes of emergency department application of the vancomycin powder in wounds with exposed bone fragments amenable to powder application without injection into the wound site; the POWDER study will directly test the concept we presented herein. The results of these studies could have clinical implications for a new antibiotic application method in the treatment of open fracture wounds.

## 7 Implications for emergency care

The morbidity burden of open fractures, especially of long bones, is high, and strongly influenced by the presence of infection. Strategies to mitigate infection risk, such as parenteral antibiotics or early surgery, are unsatisfactory as evidenced by continued high infection rates. Timing of the intervention may be crucial, as bacteria quickly set up antibiotic, surgery, and immune-resistant biofilms. An early intervention that exposes pathogenic bacteria to high concentrations of antibiotic prior to biofilm development may confer significant advantage over current approaches. Topical administration of antibiotic may in the future be shown to confer benefit. As a time-sensitive therapy it is within the scope of emergency physician practice and could easily and quickly be applied at the bedside soon after arrival in the emergency department. If studies support such use (and to be clear, we are not yet advocating for such empiric use), administration of antibiotic in the prehospital setting is a logical extension and may improve efficacy if transport times are prolonged. In military medicine and other austere environments, early application of topical antibiotics may prove significant as the technical skill needed to apply the antibiotic is likely modest, thus allowing far-forward medics and other first responders to provide the therapy.

## 8 Conclusion

Open fracture wounds are inherently difficult to manage due to immediate bacterial contamination upon injury.^[7,24]^ The ideal method for localized antibiotic delivery is described as one that can be applied quickly, prevents infection, does not require subsequent surgery for removal, does not cause system toxicity, and provides high concentrations of antibiotic to the wound site.^[45,46]^ Local antibiotic delivery methods such as PMMA cement, collagen gauze, chitosan sponge, and calcium sulfate cannot be applied until surgery, which does not allow for timely administration. A topical antibiotic powder, such as vancomycin, can be applied in high concentrations in the prehospital or emergency department setting and without the need for a delivery vehicle suggesting it could be a better treatment for open fractures. Vancomycin powder appears safe and effective on elective surgeries, and its low cost will not be a financial burden to hospitals.^[34]^ Although the results of the ongoing clinical studies are needed for a change in clinical practice to occur, this is one of the few new concepts which can be rapidly implemented in the emergency department or prehospital environment that may improve outcomes of open fractures.
